# Disease awareness and experience of adolescent depression patients: a meta-synthesis of qualitative studies

**DOI:** 10.3389/fpsyt.2025.1708827

**Published:** 2025-12-29

**Authors:** Pei Gao, Fan Guo, Yanrong Xu, Yue Wang, Liping Cui

**Affiliations:** 1 College of Nursing, Shanxi Medical University, Taiyuan, China; 2 Department of Nursing, Shanxi Bethune Hospital, Shanxi Academy of Medical Sciences, Third Hospital of Shanxi Medical University, Tongji Shanxi Hospital, Taiyuan, China

**Keywords:** adolescents, depression, awareness, experience, meta-synthesis

## Abstract

**Clinical trial registration:**

To systematically evaluate the cognition and real experience of adolescent patients with depression towards the disease, in order to comprehensively understand the cognitive and psychological predicaments they face and provide a basis for formulating targeted intervention strategies for adolescent patients with depression.

**Design:**

A systematic review and meta-synthesis of qualitative studies.

**Methods:**

Qualitative studies on the cognition and real experience of adolescent patients with depression towards the disease were retrieved from Chinese and English databases from the establishment of the databases to June 2025. The quality of the literature was evaluated using the JBI qualitative research quality evaluation standard, and the convergent meta-synthesis method was used for integration.

**Results:**

A total of 12 articles were included, and 37 research results were extracted. Two themes and nine sub-themes were identified, namely, cognition of the disease (misunderstanding of the disease, stigma towards the disease, and cognition of the prognosis of the disease), and real experience of the illness (diversity of influencing factors of the illness, somatization symptoms after the illness, emotional and psychological experiences after the illness, reconstruction of interpersonal relationships, predicaments and powerlessness in treatment, and establishment of new values).

**Conclusion:**

Teenagers with depression often have cognitive biases and undergo complex, multi-dimensional painful experiences. Healthcare professionals should first assess and restructure the patients’ cognitive biases and provide targeted treatment for their painful experiences. At the same time, scientific disease cognition and intervention strategies should be extended from hospitals to families and schools, and a collaborative support system should be established to jointly address the psychological needs of adolescent patients.

**Systematic Review Registration:**

https://www.crd.york.ac.uk/PROSPERO/view, identifier CRD420251105051.

## 1 Introduction

Depression has become a serious public health issue (1). According to the World Health Organization, over 300 million people worldwide suffer from this disease. The prevalence rate has been continuously increasing in recent years and is expected to rank first globally by 2030 (2). The core symptoms include patients losing interest in daily activities, finding past hobbies and social interactions dull, and having difficulty deriving pleasure from life (3, 4). Even more seriously, some patients may even have suicidal thoughts (5). Among this large group, teenagers account for 27% of all patients (6), making them a group that requires special attention.

The high incidence rate of depression among teenagers is closely related to their unique developmental stage. Hormonal fluctuations, the exploration of self-identity, escalating academic pressure, and the complexity of peer dynamics significantly increase the risk of depression among teenagers (7), which severely disrupts the lives, studies, and social relationships of teenagers with depression, and imposes a long-term and huge burden on the stability and development of families and society (8, 9). To effectively and promptly address this challenge, governments around the world attach great importance to it and actively introduce relevant policies to support (10, 11). However, in addition to extensive policy support, the analysis of the subjective cognition and experience of teenagers with depression determines the effectiveness of clinical treatment.

Disease awareness is a crucial psychological mechanism, which encompasses the understanding and coping strategies that adolescent patients develop when facing the threat of depression (12). This directly determines the patients’ willingness to seek help, and also affects their treatment compliance, recovery process and prognosis (13). Real experience refers to the subjective perception of a patient regarding their illness. It encompasses the emotional fluctuations, physical discomfort, and changes in interpersonal relationships that the patient undergoes during the course of the illness. Disease awareness and real experience are not independent of each other; instead, they form a cycle: the painful experience provides the basis for negative disease awareness, and negative disease awareness in turn drives the occurrence of even worse experiences, leading to the recurrence of depressive symptoms in a cycle (14). This long-term psychological and social pressure resulting from the interaction of cognition and experience can also lead to dysfunction of the hypothalamic-pituitary-adrenal (HPA) axis, releasing large amounts of stress hormones such as cortisol (15). This physiological change will further exacerbate the depressive symptoms, causing the patient to fall into a vicious physical and mental cycle. Therefore, this study focuses on the two key dimensions of disease awareness and real experience and conducts an in-depth analysis of their impact on adolescent patients with depression.

At present, numerous qualitative studies have been conducted on adolescent patients with depression both domestically and internationally (16, 17). However, the disease cognition and experiences of patients vary significantly across different regions, cultural backgrounds, and social environments, resulting in incomplete conclusions that fail to fully reflect the true inner feelings of adolescents with depression. Therefore, this study adopted a meta-integration approach to summarize and generalize the real experiences and cognition of adolescents with depression, providing a basis for the formulation of targeted intervention strategies in the future, and aiming to offer reference for the prevention and treatment of adolescent depression worldwide.

## 2 Methods

### 2.1 Search strategy and selection criteria

This systematic review and meta-analysis adhere to PRISMA guidelines. We carried out our main search in 11 databases:(1) PubMed, (2)Web of Science, (3)EMBASE, (4)CINAHL, (5)Cochrane Library, (6)Wanfang Database, (7)VIP Database, (8)CNKI, (9) PsycINFO, (10)CBM, (11)Scopus. The search period covers the period from the establishment of the database until June 2025. Searches were conducted using a combination of subject headings and free words. Search terms:”adolescents”, “teenagers”, “young people”, “boys/girls”, “minors”, “young adults”,”students”,”depression”, “anxiety”, “mental illness”,”experience”, “feeling”, “attitude”, “cognition”,”perception”,” qualitative research”, “qualitative study”, “study, qualitative”.

### 2.2 Inclusion and exclusion criteria

#### 2.2.1 Inclusion criteria

The inclusion criteria for this study followed the PICoS principle.

Participant(P):aged between 10 and 19 years old, meeting the diagnostic criteria for adolescent depression(DSM-5) (18), or explicitly described in the original text as a teenager who is undergoing depression treatment.

Interest of Phenomena(I):explore the subjective perceptions (such as views on the causes, symptoms, treatment and prognosis of depression) and/or real experiences (such as emotional feelings, changes in interpersonal relationships, academic impacts, physical symptom sensations, etc.) of teenagers regarding depression;

Context(Co):medical institutions, schools, and families;

Study Design(S):including but not limited to descriptive studies, phenomenological studies, grounded theory, etc.

#### 2.2.2 Exclusion criteria

①the research subjects also suffered from other severe mental disorders (such as schizophrenia, bipolar disorder) or major physical diseases that significantly affected their experiences (such as cancer);②non-Chinese or English literature; ③incomplete information, unable to obtain full text; ④repetitive publications or incomplete data.

### 2.3 Screening and data extraction

The retrieved literature was imported into the NoteExpress software to remove duplicate entries. Two researchers with knowledge of evidence-based medicine and who had received training in qualitative research independently conducted the literature screening and data extraction and cross-checked each other’s work. When there were inconsistencies in the results, a third researcher participated in the discussion and made the final judgment. The core information extracted included the author’s name, publication year, country, research methods, research subjects, the phenomenon under investigation, and the main research conclusions.

### 2.4 Quality appraisal

The process involved two reviewers who independently assessed the quality of the included studies using the “Quality Appraisal Standards for Qualitative Research” from the Australian Centre for Evidence-Based Healthcare (JBI) (19). Both reviewers had received training in evidence-based practice methodology. Each criterion was assessed with responses of “yes”, “no”, or “unclear”. Following the evaluation, the literature could be classified into either Grade A, B, or C. Grade A literature wholly fulfills the quality criteria, exhibiting the least probability of bias. Grade B literature partially fulfills the quality criteria, indicating a moderate probability of bias. Grade C literature does not fulfill the quality criteria at all, signifying a greater probability of bias. Ultimately, the literature of Grades A and B was incorporated, while Grade C literature was excluded.

### 2.5 Synthesis and analysis of data

During the data processing stage, the convergent integration method recommended by JBI (19) was adopted to integrate the results. This method involves extracting the main themes, potential implications, and classifications from the research conclusions, and then further summarizing and generalizing based on these contents to enhance the interpretability and comprehensiveness of the results. Eventually, various categories were merged to form highly generalized thematic results.

## 3 Results

### 3.1 The basic characteristics and quality evaluation of the included literature

**Figure 1:**
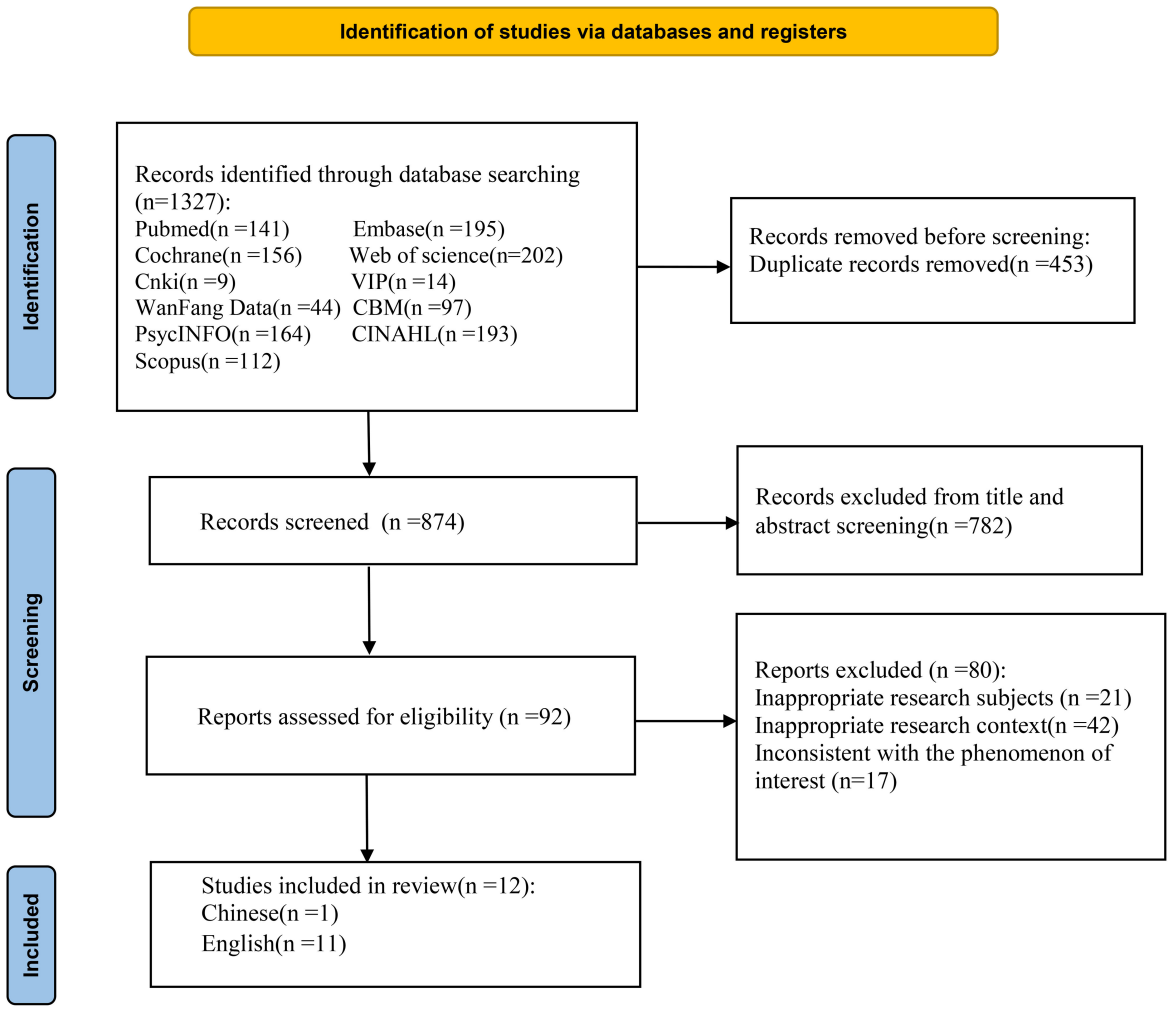
Flow diagram of the study selection process.

The initial search yielded 1327 relevant articles. Finally, 12 articles were included (20–31), including 11 English articles and 1 Chinese article (**Figure 1**). The basic characteristics of the included articles are shown in **Table 1**. Among the 12 included studies, there were a total of 292 teenagers with depression, 153 females and 93 males; their ages ranged from 10 to 19 years old. 34 participants did not specify their gender, and 4 teenagers were of the gender-neutral type. The basic characteristics of the included studies are shown in **Table 1**, and the results of the methodological quality assessment are presented in **Table 2**.

**Table 1:** The basic characteristics of the included literature (n=12).

Authors	Year	Origin	Methodology	Object	Aim	Results
Sánchez et al. (20)	2024	Germany	Phenomenological Research	Ten teenagers (seven girls and three boys, aged 14 to 17)	Exploring teenagers’ understanding of depression.	Three themes: Desiring a disconnected world; Isolating oneself; Being indifferent to everything
Wentholt et al. (21)	2024	Netherlands	Grounded theory	34 teenagers (27 girls, 7 boys, aged 11-17)	Understanding the analysis of teenagers regarding their own depressive behaviors.	Three: External environmental pressure; Changes in one’s own emotions after depression; Understanding of the disease.
Antonia et al. (22)	2024	UK.	Description of qualitative research	Twenty medical students (12 females, 7 males, 19 years old)	Describe the occurrence of depression among medical students and their understanding of depression.	Three themes: Medical culture; Fear of seeking help; Negative impact on academic life.
Sweeney et al. (23)	2024	UK.	Phenomenological Research	19 teenagers (11 girls, 5 boys, aged 14-18)	Exploring the fatigue experiences of teenagers in the context of depression and their feelings towards depression.	Two themes: Feeling persistent fatigue in depression; Shamefulness in seeking help after depression.
Zhu L et al. (24)	2024	China	Description of qualitative research	18 teenagers (11 girls, 7 boys, aged 12-18)	Exploring the causes of depression among teenagers and the corresponding coping strategies.	Three themes: The triggering factors of depression; The experience of emotional complexity during the illness; Various perspectives and understandings of depression.
Chuntana et al. (25)	2024	Thailand	Explanatory Phenomenology Research	14 teenagers (10 girls, 4 boys, aged 15-18)	Understanding the experiences and symptoms of adolescent depression.	Four themes: Striving to understand one’s own situation; Feeling depressed and withdrawing; Conflicting emotions towards self-harm; Thoughts about subsequent treatment.
Poon N Yet al. (26)	2024	Singapore	Description of qualitative research	14 teenagers (11 girls, 3 boys, aged 13-19)	Exploring the experiences and perceptions of adolescents with depression.	Three themes: Teenagers talk about their own illness experiences; Nursing experiences; Reflections after treatment.
Dardas et al. (27)	2019	Arab	Description of qualitative research	92 teenagers (56 males, 36 females, aged 14-17)	Understand the experiences of depression among teenagers, identify the perceivable influencing factors, and assess the attitudes towards depression intervention.	Two main themes and four sub-themes: Attention to depression among teenagers (symptom overview, uncertainty and the perceived causes of depression); Life experiences of depression patients (seeking support resources and avoiding resources).
Watson et.al (28).	2019	UK.	Explanatory Phenomenology Research	34 teenagers (the specific circumstances are not mentioned)	Exploring the feelings and experiences of adolescents with depression regarding the lack of pleasure.	Four themes: experiencing the loss of happiness and emotional flatness; lack of motivation and active participation; loss of connection and sense of belonging; questioning one’s self-awareness, goals and overall situation.
Shu Wan al (29).	2022	China	Phenomenological Research	20 teenagers (2 boys, 18 girls, aged 13-17)	Exploring the experiences of adolescent patients with depression and their understanding of life and death.	Two main themes and seven sub-themes: Perspectives on death (willing to think about death topics, looking forward to discussing death topics, being overly calm when talking about death topics, having cognitive biases); Perspectives on the meaning of life (derived from loving and being loved, having confusion about the sense of meaning, life being meaningless).
Katharina et al. (30)	2016	Germany	Phenomenological Research	6 teenagers (five girls, one boy, aged 15-19)	Exploring the treatment experiences and reflections of adolescents with depression.	Four themes: The pain is overwhelming; Experiences of loneliness and isolation; Efforts to understand the pain; Treatment as the ultimate solution.
Anna et al. (31)	2021	Brazil	Description of qualitative research	11 teenagers (6 girls, 5 boys, aged 14-17)	Understanding the causes of depression among teenagers and their own thoughts.	Four themes: Contradictions in social relationships; Changes in one’s own behavior; Misunderstandings about diseases; Main characteristics of emotions.

**Table 2:** Evaluation of methodological quality (n=12).

Included studies	①	②	③	④	⑤	⑥	⑦	⑧	⑨	⑩	Quality level
Sánchez et al. (20)	Y	Y	Y	Y	Y	N	N	Y	Y	Y	B
Wentholt et al. (21)	Y	Y	Y	Y	Y	N	N	Y	Y	Y	B
Antonia et al. (22)	Y	Y	Y	Y	Y	Y	N	Y	Y	Y	B
Sweeney et al. (23)	Y	Y	Y	Y	Y	Y	N	Y	Y	Y	B
Zhu L et al. (24)	Y	Y	Y	Y	Y	Y	Y	Y	Y	Y	A
Chuntana et al. (25)	Y	Y	Y	Y	Y	N	N	Y	Y	Y	B
Poon N Yet al. (26)	Y	Y	Y	Y	Y	N	N	Y	Y	Y	B
Dardas et al. (27)	Y	Y	Y	Y	Y	Y	N	Y	Y	Y	B
Rebecca et al. (28)	Y	Y	Y	Y	Y	Y	Y	Y	Y	Y	A
Shu Wan et al. (29)	Y	Y	Y	Y	Y	N	Y	Y	Y	Y	B
Katharina et al. (30)	Y	Y	Y	Y	Y	N	N	Y	Y	Y	B
Anna et al. (31)	Y	Y	Y	Y	Y	N	N	Y	Y	Y	B

Appraisal Checklist: ① Is there congruity between the stated philosophical perspective and the research methodology? ② Is there congruity between the research methodology and the research questions or objectives? ③ Is there congruity between the research methodology and the data collection methods? ④ Is there congruity between the research methodology and the representation and the data analysis? ⑤ Is there congruity between the research methodology and the interpretation of results? ⑥ Is there a statement locating the researcher culturally or theoretically? ⑦ Is the influence of the researcher on the research, and vice- versa, addressed? ⑧ Are participants and their voices adequately represented? ⑨ Is the research ethical according to current criteria or, for recent studies, and is there evidence of ethical approval by an appropriate body? ⑩ Do the conclusions drawn from the research report flow from the analysis or interpretation of the data?

Appraisal result: Y = yes; N = no; U = unclear; N/A = not applicable.

### 3.2 Meta-analysis results

After carefully reading and analyzing 12 pieces of literature, extracted a total of 37 research results. Then grouped and combined similar results into 9 new categories, and synthesized them into 2 synthesized findings.

#### 3.2.1 Synthesized finding1: cognition of diseases

Teenagers with depression usually get trapped in a vicious cycle driven by erroneous cognition. They often misinterpret their clinical symptoms as temporary mood swings, personal character flaws, or even supernatural factors. This erroneous cognition directly gives rise to and exacerbates a strong sense of internalized stigma and self-stigma, jointly constructing a pessimistic and desperate outlook on the prognosis of the disease.

##### 3.2.1.1 Category 1: misunderstandings about diseases

Most teenagers with depression do not have a clear understanding of the disease. [“I feel bad most of the day and many days of the week. I’m too tired, but I don’t know that these are the precursor symptoms of depression” (25)]; or they misunderstand depression, thinking it is just a temporary mood swing that can be overcome through self-regulation [“I have the symptoms they mentioned, but I think I can adjust myself, but the symptoms are getting worse” (27)]; and even in some countries, some teenage patients attribute the occurrence of depression to God [“Depression has no cause. When you don’t believe in God, you will feel pessimistic. God makes you lose happiness and strength” (27)].

##### 3.2.1.2 Category 2: the stigma associated with diseases

When discussing their own illness, adolescent patients often exhibit complex psychological activities [*“Everyone around me is normal, but I am a freak in others’ eyes and cannot be accepted by society”* (20)]; when recounting their illness experiences, they are filled with self-blame [*“I am always very self-critical. I am depressed and afraid that others will discover my abnormality”* (23)]; most adolescent patients are worried about possible discrimination [*“I am afraid of treatment. If I behave or talk about my true thoughts, I am afraid of being humiliated, misunderstood, and even being alienated by my family”* (27)].

##### 3.2.1.3 Category 3: understanding of disease prognosis

Diseases have a significant impact on adolescent patients, and they generally pay close attention to the development of the disease prognosis [*“After a period of treatment, the condition still doesn’t improve. I feel desperate and I want to take suicide to find relief”* (29)]; these disease symptoms may persist and worsen continuously, causing negative effects on daily life [*“My condition has completely deteriorated. I feel very bad and I want to cut my wrist to end my life”* (25)].

#### 3.2.2 Synthesized finding 2: the true experience of the illness journey

The true experience of adolescent patients with depression is a multi-dimensional and constantly changing process. It involves severe physical symptoms and extreme emotional experiences. The complex interpersonal relationships among family, school, and peers also affect the patients’ experience. The internal physical pain and external interpersonal conflicts jointly influence the patients’ attitude towards treatment and their willingness to seek help.

##### 3.2.2.1 Category 4: the diversity of factors influencing illness

The occurrence of adolescent depression is influenced by multiple factors including individual traits and environmental factors. The introverted and sensitive personality trait makes them inclined to social avoidance [*“I was not good at* sp*eaking since childhood, was afraid of approaching others, and was accustomed to immersing myself in my own world”* (31)], while family disharmony is an important factor contributing to depression [*“I didn’t g*et al*ong well with my mother, and I was extremely tired both mentally and physically”* (23)], high school pressure can also cause the occurrence of depression in adolescents [“Failing to meet academic expectations is the direct cause of my tendency to self-harm” (24)], and traumatic stress events can also have an impact on the psychology of adolescents, leading to depression [*“At school, I would encounter bullying and feel threatened”* (31)].

##### 3.2.2.2 Category 5: physical symptoms after illness

The physical symptoms exhibited by adolescents with depression vary from person to person. Some patients have relatively mild symptoms [*“I often have trouble sleeping, often feel sad, often skip classes and have headaches”* (30)]; while more patients show obvious motor inhibition [*“I don’t want to go out. It feels as if there is a heavy burden on my body. I feel numb all over”* (31)]; as the condition worsens, physical discomfort spreads from local areas to generalized pain [*“Heart pain is very uncomfortable … It has transferred to uncontrollable body pain”* (26)].

##### 3.2.2.3 Category 6: emotional and psychological experiences after illness

Adolescents with depression have complex psychological feelings, usually including low mood and persistent sadness [*“I often feel bad for no reason, or very angry”* (30)], some patients describe their inner state as completely empty [*“I have no feelings, no happiness or excitement, but no sadness either. It’s as if everything is gray”* (28)], the significant decline in these patients’ emotional regulation ability makes them prone to extreme emotional fluctuations [*“I began to find that I would be very anxious about any small matter, thus becoming depressed”* (21)].

##### 3.2.2.4 Category 7: reconfiguration of interpersonal relationships

After being diagnosed with depression, adolescents patients often experience complex and variable social relationship adjustments. In the family environment, they may feel that their parents are overly tolerant and accommodating [*“After getting sick, my parents* sp*ent more and more time with me. I made mistakes and they wouldn’t scold me, which instead made me feel even more guilty”* (27)], in peer relationships, patients often encounter social exclusion and isolation [*“During my school years, my good friends gradually drifted away from me, not playing with me anymore. I became more sensitive”* (23)]; moreover, in an environment with heavy academic pressure, their abnormal state makes the teacher-student relationship become more tense [*“In my school, the academic pressure was quite high, and the teachers were also strict. But due to my physical condition, I was unable to complete my homework, and the teachers mistakenly thought I was looking for excuses”* (24)].

##### 3.2.2.5 Category 8: challenges and feelings of powerlessness during treatment

Most adolescents with depression have a fear and avoidance mentality towards treatment during the treatment process, preferring to hide their problems rather than actively seeking external help [*“I don’t want to seek help. People can’t truly understand what I want. I prefer to hide myself “* (23)], in addition, some adolescent patients are also worried about the possible consequences of mental health diagnosis [*“I am worried about the consequences of seeking help for mental health problems. I am afraid of receiving a mental health diagnosis, which may have permanent and potential harm to my future career”* (22)].

##### 3.2.2.6 Category 9: establishment of new values

During the process of fighting against the disease, some individuals gradually establish new values and self-awareness. They begin to deeply understand their emotions and needs, and learn to face their emotions, achieving self-healing [*“In moments of being overwhelmed, desolate, and detached, the self-inflicted harm reignited my vitality, enabling me to endure the darkness of my existence”* (24)]; Key social support can also promote this transformation [*“After coming to this school, I met a very good teacher. I wanted to change myself, so I consulted a doctor, and subsequently my depression improved”* (25)].

## 4 Discussion

### 4.1 Enhance disease awareness

This study found that the majority of adolescent patients have a lack of in-depth understanding of the disease. The most prominent manifestation is disease cognition bias, a strong sense of stigma, and a pessimistic prognosis of the disease. The incorrect cognition of the disease by adolescent patients with depression also negatively affects disease coping. This incorrect cognition may stem from the fact that adolescent patients are in the stage of exploring self-identity and their minds are not yet mature, making it difficult for them to notice the early stages of depression, which is consistent with the research results of Ahuvia et al. (32); Furthermore, in some Western cultural contexts, especially in countries influenced by Christianity or Islam, adolescent patients may exhibit a refusal to seek medical treatment and hide the physical discomfort caused by the disease. This stems from their tendency to associate the disease with religious beliefs, viewing depression as a “soul disease” (33), and this concept may ultimately interfere with the patients’ disease cognition and treatment decisions (34). This duration of untreated illness caused by cognitive bias and stigma directly affects clinical assessment and diagnosis and hinders the effectiveness of treatment (35). Therefore, healthcare professionals should shift the focus of treatment for adolescent patients with depression from symptoms to cognition. They should first assess and intervene in the patients’ disease cognition ability (36). Healthcare professionals can use methods such as interviews to explore patients’ attributions of the disease, perception of consequences, and personal control, accurately identifying cognitive biases. In addition, corresponding psychological education, such as cognitive behavioral therapy (CBT) (37), can be used to help patients restructure their cognition and reduce their fear of social stigmatization, providing patients with a new and scientific cognitive framework. Meanwhile, when clinical practitioners conduct CBT cognitive restructuring for patients, they should also assess the impact of family members on the patient’s cognition and include family members in the treatment when necessary.

### 4.2 Face the real experience directly

The experience of adolescent patients with depression is complex and diverse. On one hand, it stems from their own painful experiences after the onset of the disease, including the factors that led to the illness, the physical symptoms after the illness, and the emotional and psychological experiences after the illness; on the other hand, it involves interactions with the external world after the illness, including the reconfiguration of interpersonal relationships, the difficulties and powerlessness during treatment, and the establishment of new values. Numerous scholars’ research (38) has shown that when adolescent patients are confronted with significant physical and mental pain, they tend to internalize the pain and are reluctant to express their thoughts. This kind of long-term internalized psychological stress can be regarded as a key trigger for the dysfunction of the HPA axis (39). This provides a possible physiological mechanism for the continuous release of hormones such as cortisol and may cause patients to fall into a vicious cycle between physiology and psychology. Therefore, healthcare professionals should first break this internalized cycle and encourage adolescent patients to externalize their inner pain and actively express it. Narrative Therapy (NT) (40), as a postmodern psychological treatment method, provides a way for this situation. It guides adolescent patients to view depression as an entity that needs to be jointly overcome, helps them break free from self-pressure, and enables them to express their stress and re-examine their predicaments. Moreover, for the intense emotional problems encountered by adolescent patients, healthcare professionals can adopt Acceptance and Commitment Therapy (ACT) (41). By using cognitive defusion and value clarification, it can reduce patients’ avoidance of negative emotions and enhance their drive for new values. Healthcare professionals can employ ACT and other mindfulness exercises to guide adolescent patients to bravely face the disease, reduce the pain caused by emotional avoidance, and enhance their psychological resilience.

### 4.3 Enhance social support systems

From the perspective of the entire study, the disease cognition and experience of adolescent patients with depression are not caused by a single factor but are the result of the joint efforts of various aspects such as family, school and society. The multiple pressures from family, school and society, along with the complex environment, have led to a high incidence of depression among adolescents. Yim et al. (42) found that adolescent patients in an Eastern cultural background are deeply influenced by collectivist values, and they are more likely to believe that mental illness is not only an individual problem but also a disgrace for the entire family. Therefore, while healthcare professionals are conducting mental health education for patients, they must also provide corresponding psychological training to family members, changing their attitudes and perceptions, in order to reduce the stigma associated with mental illnesses within the family (43). Additionally, significant academic pressure and tense peer relationships are also core stressors commonly faced by adolescent patients. Strengthening the psychological health training for teachers can enhance their knowledge of the disease and their ability to identify it early. This helps create a stigma-free and supportive environment on campus, directly improving the daily experiences and negative disease perceptions of patients.

Moreover, research has shown that social support is an effective protective factor in preventing the occurrence and development of depressive symptoms in adolescents (44). Utilizing social media to promote anti-stigma social campaigns and improving the social security mechanism can reduce the psychological help-seeking costs for adolescent patients and provide them with an external environment that truly understands and accepts them. Therefore, it is urgent to establish a family-school-social linkage support system to better address depression issues.

## 5 Limitations of the review

This study strictly followed the JBI integration method, and the quality assessment standards set by JBI for qualitative research, aiming to conduct a qualitative evidence synthesis analysis of the disease cognition and actual experiences of adolescent patients with depression. However, this study undoubtedly has certain limitations. Firstly, the final included research literature was only 12 articles, and grey literature was not retrieved and included, which may lead to an incomplete range of included literature; in addition, the included research articles had different cultural backgrounds, social conditions, and policy factors, which may limit the general applicability of the research results. At the same time, most of the literature failed to clearly present the cultural and value viewpoints of the researchers, which may introduce certain biases. Finally, since the meta-analysis is based on the analysis of the included studies to draw conclusions, the reliability of the comprehensive results is inevitably constrained by the quality of the original studies. Therefore, in future research, more high-quality original studies should be explored to ensure the quality of the articles.

## 6 Conclusion

This study adopted the JBI qualitative systematic review method and integrated 12 studies. Eventually, two core results were summarized. The research results indicate that adolescent patients with depression have complex and diverse perceptions and experiences of the disease, and both of these factors jointly affect the patients’ enthusiasm for disease treatment. At the same time, when facing the disease, adolescent patients are significantly influenced by their families and schools. Healthcare professionals should enhance the psychological knowledge training for parents and school teachers, detect and handle the disease at an early stage, and all countries should also improve the social support system and social security mechanism to safeguard the healthy growth of adolescents. In future research, more diverse and extensive qualitative studies should be included to further explore the dynamic evolution process of the disease experience of adolescent patients with depression, and to promote the healthy development of adolescents’ mental health. At the same time, more personalized intervention strategies should be developed for adolescent patients, in order to make a more substantive contribution to promoting the physical and mental health development of adolescents.

## Data Availability

The original contributions presented in the study are included in the article/**Supplementary Material**. Further inquiries can be directed to the corresponding authors.
