# Supplementary material for: Disease awareness and experience of adolescent depression patients: a meta-synthesis of qualitative studies
Source: Front Psychiatry. 2025 Dec 29;16:1708827. doi: 10.3389/fpsyt.2025.1708827 (PMC12791038; doi:10.3389/fpsyt.2025.1708827)
Supplement: Supplementary file 1 [file Supplementaryfile1.docx]

Supplementary files

**The results are partly sourced from:**

*References 20: A phenomenologically grounded specification of varieties of adolescent depression.*

**original text :**

1. Mode speciffer described these feelings particularly in relation to their peers. Participant #7, for instance, said, “I cannot really associate with them …, it seems very strange to me to talk to people my age …. They make me feel the least understood, […] the furthest away.”

2. He following description of participant #6 succinctly reffects this feeling: “Well, you just do not feel right here anymore. So, I do not know what it is …, you just do not feel cared for anymore.” This feeling of not being an object of care/love—which can take the form of a feeling of having been neglected, disrespected, or offended in some usually unclear way—is associated with the declining attitude.

*References 21:A qualitative, multi-perspective study on causal beliefs about adolescent depression.*

**original text :**

1. The theme ‘Characteristics of the child’ consisted of statements about characteristics that are related to the child('s personality) and are present across contexts; ranging from specific statements like ‘he has always been sensitive, which might make him more susceptible to having a hard time […]

*References 22:**The experiences of students with mental health difficulties at medical school: a qualitative interview study.*

**original text :**

1. I just didn’t want that label. I didn’t want to be a medical student who had a condition, it just felt really like ‘oh I don’t want to have that on there, and I just kept worrying about . . . because as I got to third and fourth year I knew that I really liked things like GP and psychiatry so I kept thinking well I don’t think anyone would want a GP or psychiatrist who’s got this thing going on that they call a disability or that needs medication, so it was kind of a problem of accepting it in that kind of way. (Participant 2)

*References 23 : "I'm always going to be tired": a qualitative exploration of adolescents' experiences of fatigue in depression.*

**original text :**

1.As Ben described: “I find when I'm mentally drained mostly I feel physically weak um I don't know, like biologically if I am actually, you know, if my muscles are actually tired”.

2.“All my relationships declined ‘cause I’d just be very crabby and snappy erm and I never hung out with my friends ‘cause I was too tired so I wouldn’t go out.” (Megan)

3.Adolescents felt that this lack of understanding stemmed from the fact that their fatigue was related to depression and did not have an identifable physical cause. Without tangible evidence of an illness, friends and family struggled to understand where this tiredness came from, and why it seemed to have such a signifcant impact, leading to it not being taken seriously. If you have like a physical thing people’ll understand why you’re tired after you’ve broke your arm ‘cause your body wants to repair itself, but people didn’t really understand why I was tired because I was just depressed.” (Megan)

*References 24:Experiences and Cognitive Characteristics of Non-Suicidal Self-Injury in Adolescents With Depression: A Qualitative Study.*

**original text :**

1. Many adolescents report that when negative emotions accumulate to an unbearable level, they off en seek immediate emotional relief through self-injury: “Before I resort to self-harm, I am overwhelmed with negative feelings” (Participant [P] 7); “I am justtoo miserable” (P8).

2. For those attempting to break free from this pattern, the challenge is formidable:

Controlling these overwhelming mental and physical urges seems impossible.

3. School Factors. School climate, teacher–student interactions, and peer relationships have a profound impact on adolescents’ self-injurious behaviors. Some adolescents experienced humiliation and bullying among classmates, heavy academic loads, and bad peer inff uences. TT ese factors not only put pressure on their mental health, but may also prompt them to resort to self-harm to cope with these pressures.

*References 25:The experience of Thai adolescents with depression: A qualitative study.*

**original text :**

1. I don't know my situation, it is depression or not, I feel bad most of day and many days in a week. Nobody understand me, I cannot tell everyone. It is so bad. (Interviewee 2)

2.When I felt bad, sometimes…. I slit my wrist (interviewee 12)

3.Because of being depressed I have made really stupid choices and done some stuff that's given me a really bad reputation. I started to drink alcohol with strangers. Sometimes, I cracked my skull when I have no idea. (Interviewee 7)

*References 26: Understanding adolescent depression in Singapore: a qualitative study.*

**original text :**

1. One participant said, “Heart pain very uncomfortable … transfer to physical pain that can be controlled”.

2. Five (36%) participants encountered more unique stressful situations. This included one participant who struggled with being transgender and was uncomfortable with the physical changes of puberty and another who have had chronic back pain that prevented her from pursuing her passion in sports. One participant described feeling guilty about failing to resuscitate a man with a heart attack.

*References 27:Depression in Arab Adolescents: A Qualitative Study.*

**original text :**

1.Further analysis grouped these causes of depression across three levels: individual, family, and school. At the individual level, most participants believed that a lack of faith in God and God’s will predispose an individual to depression.

2.Participants expressed concerns over being ashamed, misunderstood, or even estranged from their families and tribe if they showed or talked about their real emotions. Female participants reported fears that their communities would think they were bewitched, and this might exacerbate their depression and hurt them further. Few participants reported that depression is considered a dangerous and difﬁ cult to treat disease.

*References 28:Understanding anhedonia: a qualitative study exploring loss of interest and pleasure in adolescent depression.*

**original text :**

1. Yeah like although they were the things I enjoyed, although I knew I should be enjoying them, for some reason like, I just like wouldn’t have the motivation to do it.” (Ivy, clinic).

*References 29:A Qualitative Study on the Cognition of Life and Death among Adolescents with Depression and Self-Injurious Behaviors*

**original text :**

Interviewee 5: "Life is like a flower. Death is when consciousness is gone and disappears. I think everything vanishes then. When you die, you feel nothing at all." Interviewee 20: "Actually, I don't think death is a big deal."

*References 30:The Experience of Depression: A Qualitative Study of Adolescents With Depression Entering Psychotherapy.*

**original text :**

1. YP all spoke of the core symptoms of depression as described in the psychiatric literature, including depressed mood, loss of interest, fatigue, trouble concentrating, sleeping problems, and so on, but throughout their narratives, a strong sense of suffering and burden was prevalent.

2.Whereas for Melissa, the aggression seemed to come out of nowhere: And also often, that I simply (.) without any reason get in a bad mood or I am totally annoyed.

*References 31:Social isolation as a core feature of adolescent depression: a qualitative study in Porto Alegre, Brazil.*

**original text :**

1.I don’t know if this is specific to Brazil, but bullying is one of the main causes. The person is already facing a lot, and on top of that, suffers bullying. The adolescent wants to be part of a group, and when they face bullying, they feel threatened.” (Adolescent 1, female, 14)

2. While the number of participants in our study was similar across both genders (6 girls and 5 boys), studies carried out in WEIRD countries identified social withdrawalas more common for boys and loneliness for girls . This may be due, however, to the fact that our study was not designed to explore loneliness as an experience per se. It is also worth noticing that experiences of loneliness may vary in different demographic and cultural groups.

3.One adolescent—#6, a 15-year-old girl with a long history of mental health issues, provided a clear picture about this component of adolescent depression:

“I want to be alone.” The first time [first depressive episode], all I could think was that I wanted to be alone, wanted to be in my own corner. All I could think about . . . I want to be alone.” (Adolescent 6, female, 15)
